# Boards of Directors: Assessing Their Functioning and Validation of a Multi-Dimensional Measure

**DOI:** 10.3389/fpsyg.2018.02425

**Published:** 2018-12-04

**Authors:** Shamiran Asahak, Simon L. Albrecht, Marcele De Sanctis, Nicholas S. Barnett

**Affiliations:** ^1^Arup, Melbourne, VIC, Australia; ^2^School of Psychology, Deakin University, Melbourne, VIC, Australia; ^3^Centre for Leadership Advantage, Melbourne, VIC, Australia; ^4^Insync, Melbourne, VIC, Australia

**Keywords:** board of directors, board of directors effectiveness, measures of effectiveness, validity and reliability of dimensions board functioning, validity and reliability of dimensions of board effectiveness

## Abstract

Boards of Directors that function effectively have been shown to be associated with successful organizational performance. Although a number of measures of Board functioning have been proposed, very little research has been conducted to establish the validity and reliability of dimensions of Board performance. The aim of the current study was to validate the measurement properties of a widely-used model and measure of Board performance. Exploratory Factor Analysis (EFA) and Confirmatory Factor Analysis (CFA) were conducted on online survey data collected from 1,546 board members from a range of Australian organizations. The analyses yielded 11 reliable factors: (1) effective internal communication and teamworking (2) effective leadership by the Chair (3) effective committee leadership and management (4) effective meeting management and record keeping, (5) effective information management (6) effective self-assessment of board functioning (7) effective internal performance management of board members (8) clarity of board member roles and responsibilities, (9) risk and compliance management (10) oversight of strategic direction, and (11) remuneration management. These dimensions to a large extent correspond to previously suggested, but not widely tested, categories of effective Board performance. Despite self-reported data and a cross-sectional design, tests of common method variance did not suggest substantive method effects. The research makes significant contributions to the corporate governance literature through empirical validation of a measure shown to reliably assess 11 discrete dimensions of Board functioning and performance. Practical and theoretical implications, study limitations and future research considerations are presented.

## Introduction

One of the most important factors underpinning successful organizational performance is the functioning of its board of directors (Machold and Farquhar, 2013; Bezemer et al., 2014). Boards of directors are responsible for the oversight of systems and processes that direct, control, and govern an organization's strategy, leadership decisions, regulatory compliance, and overall performance (Mowbray, 2014). Although board effectiveness has often been evaluated in terms of financial metrics such as return to shareholders, return on investment or return on assets (Dalton et al., 1998; Erhardt et al., 2003; Carter et al., 2010), effective boards also effectively oversee and challenge management's strategic, compliance, and operational decisions (Orser, 2000; Babić et al., 2011). Effective corporate governance also extends to framing, setting and monitoring an organization's values and culture (Adams, 2003; Ritchie and Kolodinsky, 2003), and ensuring that business decisions and practices are conducted ethically, fairly, and comply with community and regulatory standards (Nicholson and Kiel, 2004; Brown, 2005).

The responsibility for a large number of corporate failures, scandals, and ineffective business transformations has been attributed to ineffective board functioning (Carpenter et al., 2003; Thomson, 2010). The collapse of Lehman Brothers during the global financial crisis in 2008 is an often-cited example of how an ineffective board failed to ensure appropriate risk settings and policies to monitor corporate practice (Kirkpatrick, 2009). The press and regulatory authorities from across the world continue to expose corporate scandals (e.g., Wells Fargo Bank, Volkswagen, Australian Mutual Provident Society) that have been attributed to Board mismanagement and ineffectiveness (e.g., Cossin and Caballero, 2016).

### Conceptualizing and Measuring Board Functioning

Given corporate sensitivities to the potential impact of less than flattering information about board functioning being made available to external stakeholders, it has been ‘extremely difficult for researchers to measure the task performance of boards in ways that are both reliable and comprehensive’ (Forbes and Milliken, 1999, p. 492). In practice, most boards use self-developed or consultant developed check-lists to self-assess the effectiveness of the Chair, the board, and board members over a range of composition, procedural, group process and performance factors (Nicholson and Kiel, 2004; Chen et al., 2008). However, very little empirical research has been conducted aimed at identifying and statistically validating the different dimensions of board functioning.

Among the existing frameworks developed to understand board behavior, Bradshaw et al. (1992) proposed that board functioning is comprised of three key dimensions: structure, process and performance. Nicholson and Kiel's 2004 framework consists of inputs (e.g., company history, legal constraints, environmental feedback), intellectual capital (e.g., knowledge, skills, industry experience, external networks, internal relationships, board culture and norms) and board roles and behaviors (e.g., controlling, monitoring, advising, resourcing) that dynamically interact to influence organizational performance. Nicholson and Kiel argued that all components and sub-components of their model need to be in alignment for boards to operate and perform effectively. Along similar lines, Beck and Watson's 2011 maturity model proposes that board effectiveness be assessed in terms of board competencies (e.g., knowledge and skills), board structures (e.g., policies, processes and procedures), and board behavior (e.g., norms, board-management relations, demonstrated values).

In terms of how board processes and functioning are measured and evaluated, a number of performance indices have been proposed and developed. Brown (2005), for example, recommended self-report surveys to evaluate individual performance behaviors such as attendance, the quality of that attendance (i.e., coming prepared to board meetings), the constructive contribution of board members (i.e., to conversations and the business of the board), and the extent to which board members have the necessary knowledge and skills to perform their roles. Brown (2005) also recommended that Board members evaluate overall board performance and organizational performance. Similarly, Beck and Watson suggested that board members self-rate their performance over a number of dimensions to determine whether their stage of maturity could best be described as “baseline,” “developing,” “consistent,” “continuous learning,” or “leading practice.” Neither of the self-assessment surveys proposed by Brown and by Beck and Wilson has been tested for measurement reliability and validity.

Leblanc and Gillies (2003, 2005), based on qualitative research, published a widely used model and measure of board functioning. According to Leblanc and Gillies (2003), an effective board “… needs to have the *right* board structure, [be] supported by the *right* board membership, and engaged in the *right* board processes” (p. 9). As such LeBlanc and Gillies' framework suggests that effective boards are defined by what the board does (roles), who is on the board (composition), how the board operates (structure and processes), and the direction the organization takes as a result of advice from the board (strategy and planning). Leblanc and Gillies' (2005) measure of board functioning consists of 120 statements that board members rate to derive scores for 10 scales or sub-scales represented in their model. Board structure and board composition are measured as unidimensional 12-item scales. Board processes are measured by five 12-item sub-scales: leadership by the Chair; board member behavior and dynamics; board and management relationships; meeting management and processes; and, information management and internal reporting. Board tasks are measured by three 12-item sub-scales: direction, strategy and planning; CEO, organizational performance and compensation; and, risk assurance and external communication. Although widely used, the measurement properties of the measure have not been widely validated. As such, the present research aims to apply validation processes to a measure of board effectiveness and to therefore establish psychometrically defensible dimensions of board functioning that can be used to reliably evaluate board performance.

## Method

### Participants and Sampling Procedure

A large Australian consulting firm specializing in survey research and consulting collected 9 years of board self-evaluation data using a modified version of Leblanc and Gillies' (2005) Board Effectiveness Survey. The data were collected for the purposes of measuring board effectiveness for clients, internal research, norming and marketing. Participants were emailed invitations to complete the survey online. Participants were informed that the survey was anonymous and that the data they provided could be used by the consulting firm for consulting and research purposes. Use of the data was approved by the second author's university ethics committee. The approval was granted in accord with the Australian Government National Statement on Ethical Conduct in Human Research (2007) and on the basis that the research involves the use of existing and non-identifiable data collected with no foreseeable risk of harm or discomfort for participants.

The sample consisted of 1,546 board members from a variety of industries; 73% male and 24% female. Ages of respondents varied, with 45% ranging from 55 to 64 years of age, 34% from 45 to 55 years, 12% from 35 to 44 years, 7% being 64 years and older, and 2% being 35 years and younger. Participants were members of an audit committee (37%), a remuneration committee (22%), a nominations committee (13%), or an “other” committee (28%). Experience as a board member ranged from more than 9 years (54%), 5–9 years (26%), 2–4 years (14%), and 1 year or less (6%). Most respondents (86%) were non-executive directors (i.e., no executive role in the organization and serving as independent directors), and the remaining 14% were executive directors.

### Measures

As previously noted, Leblanc and Gillies' (2005) Board Effectiveness Survey is proposed to measure four broad dimensions of board functioning: board structure and role clarity (“*what* the board is structured as”); board composition (“*who* is on the board”); board process (i.e., “*how* the board works”); and board tasks (i.e., “what the board *does*”). The measure consists of ten scales or sub-scales—(1) board structure (12 items); (2) board composition (12 items); board processes (five 12-item sub-scales)—(3) board and committee leadership, (4) board member behavior and dynamics, (5) board and management relationship, (6) meetings, agenda and minutes, (7) information and internal reporting; board tasks (three 12-item sub-scales)—(8) direction, strategy and planning, (9) CEO, organizational performance and compensation, and, (10) risk, assurance and external communication. The data contained no outcomes measures (e.g., self-ratings of Board performance; employee ratings of Board effectiveness) and did not enable links with objective organizational performance (e.g., share price, reputational indices, etc.). As such, the external validity of the measures could not be assessed.

Participants were asked to ‘Please indicate the extent to which you agree with each of the following statements’, with reference to the 120 survey statements, using a 7-point Likert scale, ranging from *(1) strongly disagree* to *(7) strongly agree*. Example items are shown in the results section.

## Results

### Exploratory Factor Analysis

Given the adapted version of the survey has not previously been validated, the data were randomly split into roughly equal halves so the results of the Exploratory Factor Analysis (EFA) conducted on one half of the data could be cross-validated with Confirmatory Factor Analysis (CFA) using the other half of the data (Tabachnick and Fidell, 2014). The Kaiser-Meyer-Olkin (KMO) measure verified sampling adequacy for the EFA (KMO = 0.924) and Bartlett's Test of Sphericity [χ^2^_(3, 745)_ = 55, *p* < 0.001] supported the factorability of the data. The EFA (*n* = 842), with maximum likelihood (ML) extraction and oblimin rotation, yielded 14 factors explaining 56% of the variance. Given that a minimum of three items is needed to reliably define a construct (Bagozzi and Yi, 1988; Jöreskog and Sörbom, 1993), the items for factors that had < 3 items were not retained for further analyses. Similarly, items that had loadings < 0.3 or that cross-loaded across factors >0.3 were not retained for subsequent analyses (Tabachnick and Fidell, 2014).

Table 1 shows that after deleting three two-item factors, items loading lower than 0.3, and three cross-loading items, a subsequent EFA yielded 11 relatively clean factors, with no cross loading items, explaining 58% of the variance. The 11 factor solution consisted of: effective board communication, teamwork and dynamics; board self-assessment; effective leadership by the chair; remuneration management; committee chair leadership and management; meeting management; information management; risk and compliance management; role clarity; strategic oversight and direction; and performance management of board members. The 11 factors, to a large extent, corresponded to Leblanc and Gillies' (2005) “how,” “do,” and “what” factors. Six of the 11 factors were comprised of exclusively or predominantly “how” items, identifying discrete process factors that suggest how a board can best operate effectively. Three of the factors were exclusively or predominantly “do” factors; one was a “what” factor, and one consisted of a who, what and a how item. The analysis did not extract a clear “who” factor.

**Table 1:** Factor loadings for the 11-factor model (ML extraction with oblimin rotation).

**Item**	**Factor 1**	**Factor 2**	**Factor 3**	**Factor 4**	**Factor 5**	**Factor 6**	**Factor 7**	**Factor 8**	**Factor 9**	**Factor 10**	**Factor 11**
How_AS	−0.736										
How_AB	−0.734										
How_AM	−0.643										
How_Y		0.752									
How_O		0.715									
Who_D		0.695									
How_V			0.751								
How_J			0.614								
How_M			0.611								
Do_AI				0.874							
Do_AB				0.860							
Do_Z				0.627							
How_AT					0.581						
How_T					0.574						
What_K					0.541						
How_X					0.459						
How_Z					0.422						
How_AV						−0.903					
How_BB						−0.536					
How_AW						−0.529					
Do_D							0.589				
How_K							0.587				
How_R							0.542				
How_C							0.500				
Do_I								−0.789			
How_AK								−0.695			
Do_AH								−0.590			
Do_U								−0.537			
Do_E								−0.517			
What_A									0.623		
What_B									0.569		
What_F									0.467		
Do_W										−0.797	
Do_X										−0.573	
Do_AF										−0.454	
Who											0.433
What											0.391
How_AZ											0.331

*n = 842*.

### Confirmatory Factor Analysis

The generalizability of the EFA solution was tested with CFA using the second half of the data (*n* = 704). As per recommendations by Kline (2011), model fit was evaluated using a range of fit indices: chi-square, chi-square to degrees of freedom ratio (χ^2^/df), comparative fit index (CFI), Tucker-Lewis index (TLI), normed fit index (NFI), root mean square error of approximation (RMSEA), RMSEA confidence intervals, and *p* of close fit (PCLOSE). Researchers (Browne et al., 1993; Hu and Bentler, 1999; Schreiber et al., 2006) have suggested cutoff levels for determining model fit: χ^2^/*df* ≤ 2, CFI > 0.95, TLI > 0.95, NFI > 0.95, RMSEA < 0.05 with RMSEA *CI* ≤ 0.06 and PCLOSE > 0.05. However, NFI, CFI and TLI values between 0.90 and 0.95 have also been argued to demonstrate good fit (Marsh et al., 1996). Global, or absolute, measures of fit such as Akaike's Information Criterion (AIC), Bayesian Information Criterion (BIC), and Consistent Akaike's Information Criterion (AIC) have also been used to compare non-nested models from the same sample that differ in complexity (Burnham and Anderson, 2002). Smaller AIC, BIC, and CAIC values suggest better fit.

As shown in Table 2, the proposed model, derived from the EFA, yielded acceptable fit: χ^2^ ratio = 2.429, TLI = 0.933; CFI = 0.942; NFI = 0.906; RMSEA = 0.045 (0.042–0.048); PCLOSE = 0.997. All standardized loadings (ranging between 0.43 and 0.88) were significant (*p* < 0.001), and only four of the items had loadings < 0.70. Table 2 also shows that a one-factor model, included for comparison purposes, did not yield acceptable fit. Example items and associated factor loadings are shown in Table 3.

**Table 2:** Fit indices for null, proposed, one-factor, and common method factor models (*n* = 704).

**Model**	**χ^2^**	***df***	**χ^2^/*df***	**CFI**	**TLI**	**NFI**	**RMSEA**	**RMSEA CI**	**P CLOSE**
Null	20542.55	1035	19.84	0.000	0.000	0.000	0.164	0.162–0.166	0.000
Proposed	1481.600	610	2.429	0.942	0.933	0.906	0.045	0.042–0.048	0.997
One-factor	6380.144	666	9.580	0.622	0.601	0.596	0.110	0.108–0.113	0.000
Commmethod	1306.498	573	2.280	0.951	0.940	0.917	0.043	0.040–0.046	1.00

*RMSEA CI, RMSEA 90% confidence intervals; commMethod, Common method factor*.

**Table 3:** Example items and loadings of the 11-factor respecified model.

**Scale**	Item^*^	**Loading**
**COMMUNICATION AND TEAMWORK**		
How_AS	Our Board works constructively as a team (i.e., through collegial, productive working relationships that foster trust and respect).	0.873
How_AB	Directors communicate well (i.e., by listening, respecting, acknowledging and building on the views and perspectives of colleagues).	0.826
How_AM	Boardroom discussions are constructive (i.e., Directors disagree without being disagreeable, assumptions are constructively challenged, views are skillfully explored….).	0.802
**BOARD SELF-ASSESSMENT**		
How_Y	We conduct a comprehensive assessment of the effectiveness of our Board Chair.	0.849
How_O	We conduct a comprehensive assessment of the effectiveness of each of our Board Committee Chairs.	0.794
Who_D	We conduct a comprehensive assessment of the effectiveness of each of our Directors.	0.781
**LEADERSHIP BY THE CHAIR**		
How_V	Our Board Chair sets a good example for me (i.e., sets, inspires and holds Directors accountable to high standards).	0.809
How_J	Our Board Chair conducts an effective decision making process (i.e., ensures that alternatives are generated…)	0.876
How_M	Our Board Chair builds healthy Boardroom dynamics (i.e., relates well with Directors and Management, deals effectively with dissent, works constructively toward consensus).	0.875
**REMUNERATION MANAGEMENT**		
Do_AI	Our CEO's remuneration package is appropriate (i.e., well structured, based on clearly documented financial and non-financial performance criteria and takes into account the responsibilities of the role, the market and the purpose of our Organization).	0.798
Do_AB	The remuneration packages of our management (other than CEO) are appropriate (i.e., well structured, based on clearly documented financial and non-financial performance criteria, …).	0.875
Do_Z	The remuneration of Management is linked to the successful implementation of our organization's strategy (i.e., strategic plan, business plan and budget).	0.696
**COMMITTEE LEADERSHIP AND MANAGEMENT**		
How_AT	The Chairs of our Board Committees are effective in communicating with our Board (e.g., ….).	0.766
How_T	Our Board Committee Chairs carry out their roles well ….	0.767
What_K	Our Board Committees …are used to effectively support the work of the Board …	0.745
How_X	Item cannot be reproduced due to copyright.	0.738
How_Z	Item cannot be reproduced due to copyright.	0.658
**MEETING MANAGEMENT (AGENDA, RECORD KEEPING, ETC.)**		
How_AW	The minutes of our Board Meetings are well managed (i.e., drafting is clear, accurate, consistent, timely and with appropriate detail).	0.679
How_BB	The written reports (including recommendations) from the Committee meetings are effective …	0.707
How_AV	The minutes of our Board meetings are well managed …	0.799
**INFORMATION MANAGEMENT**		
Do_D	Strategic issues are presented to the Board with adequate time for reflective thought (including enabling off-line communication among Directors and effective questioning of Management during Board meetings).	0.698
How_K	The information received by our Board is appropriate given the decisions we need to make …	0.838
How_R	Our … requests for information are handled well by Management …	0.735
How_C	Item not reproduced	0.742
**RISK MANAGEMENT AND COMPLIANCE**		
Do_I	Our Organization has an effective (e.g., comprehensive and integrated) enterprise risk management system (i.e., which responds to, identifies, evaluates, monitors, controls and mitigates significant risks to our Organization).	0.805
How_AK	Our Board receives appropriate information on how our organization's risks are managed ….	0.831
Do_AH	Our Board and Management have an agreed view on our organization's risk appetite …	0.769
Do_U	Item not reproduced	0.723
Do_E	Item not reproduced	0.702
**ROLE CLARITY**		
What_A	Our Board has appropriate (i.e., detailed, clear and up-to-date) documentation of the role and responsibilities of the Chair of the Board (e.g., a position description).	0.772
What_B	Our Board has appropriate … documentation of its role and responsibilities (e.g., board guidelines or Charter).	0.744
What_F	Our Board has appropriate (i.e., detailed, clear and up-to-date) documentation of the role and responsibilities of our Committee Chairs (e.g., position descriptions).	0.764
**STRATEGY OVERSIGHT AND DIRECTION**		
Do_W	Our Board approves the strategic plan only after conducting a rigorous review (including considered Board input) of the plan.	0.812
Do_X	Our Board has a full understanding of what actions are required to execute our organization's strategic plan successfully.	0.874
Do_AF	Our Board sets the broad parameters for Management's preparation of our organization's strategic plan.	0.640
**PERFORMANCE MANAGEMENT OF THE BOARD**		
Who	Inadequate performance (including inadequate commitment) by Directors is addressed promptly through peer-remediation or by our Chair taking appropriate action.	0.776
What	Our Board committees act independently of management.	0.730
How_AZ	Appropriate action would be taken if we had undesirable Director behavior (i.e., by our Board or our Board Chair).	0.715

* *All items copyright and not to be used without permission. © Board Benchmarking Australia Pty Ltd*.

Given that the data were self-report data, procedures for testing common method variance (CMV) were conducted (Podsakoff et al., 2012). As such, the fit of the proposed model was compared to the fit of a CMV model whereby parameters from an additional common method factor were specified to load on each of the items. Although Table 2 show that the fit statistics for the CMV model were marginally better than for the proposed model, the global fit indices (AIC, BIC and CAIC) showed that the fit of the proposed model (AIC = 1743.598, BIC = 2340.536, CAIC = 2471.536) was better than the fit of the CMV model (AIC = 1642.498, BIC = 2408.037, CAIC = 2576.037). Global indices are particularly useful when comparing non-nested models from the same data set (Browne and Cudeck, 1989; Raftery, 1995). Furthermore, the standardized loadings for only two of the items decreased more than 0.16 when the common method factor was included in the model. The two items were “How_O” and “Who-D,” both being “Board Self-Assessment” items (see Table 3), with decreases of 0.42 and 0.35, respectively. Otherwise, given that the standardized loading decreased, on average, a very modest 0.07 across the 38 items, and that all factor loadings remained statistically significant (*p* < 0.001) after the inclusion of the common method factor, the influence of method effects can, to a large extent, be discounted (Elangovan and Xie, 2000; Johnson et al., 2011; Podsakoff et al., 2012).

The means, standard deviations, correlation coefficients and Cronbach's alpha reliabilities for the proposed measurement model are presented in Table 4. For comparison purposes, the means, standard deviations, alpha reliabilities, and correlation coefficients derived from the EFA sub-sample (*n* = 842) are also provided. Table 4 shows the means, standard deviations, alphas, and correlation coefficients across both sub-samples were very similar. With the exception of “board performance management” (α^1^ = 0.59; α^2^ = 0.65) and “meeting management” (α^1^ = 0.74, α^2^ = 0.76), all constructs yielded excellent internal reliability, with alphas ranging from 0.80 to 0.89 (Nunnally, 1978). Additionally, none of the correlations among the 11 factors (ranging from *r* = 0.25–0.64) exceeded 0.70, and therefore do not suggest the presence of any higher-order factors (Christiansen et al., 1996).

**Table 4:** Means, standard deviations, internal consistencies, Cronbach's alpha in italics on diagonal) and correlations among the first-order variables in the re-specified model for the EFA validation sample (*n* = 842) and the CFA cross-validation sub-sample (*n* = 704).

**Variable**	Mean^a*^	*SD*^a*^	Alpha^a*^	Mean^b*^	*SD*^b*^	Alpha^b*^	**1**	**2**	**3**	**4**	**5**	**6**	**7**	**8**	**9**	**10**	**11**
Comms	5.98	0.85	0.85	5.91	0.90	0.87	–	0.29^**^	0.56^**^	0.27^**^	0.54^**^	0.45^**^	0.43^**^	0.46^**^	0.25^**^	0.51^**^	0.56^**^
SelfAssess	4.09	1.31	0.85	4.04	1.34	0.84	0.29^**^	–	0.36^**^	0.32^**^	0.42^**^	0.30^**^	0.32^**^	0.42^**^	0.53^**^	0.39^**^	0.43^**^
ChairLdshp	6.05	0.87	0.87	5.96	0.96	0.89	0.61^**^	0.38^**^	–	0.30^**^	0.42^**^	0.48^**^	0.48^**^	0.39^**^	0.33^**^	0.44^**^	0.56^**^
RemMgt	5.26	1.09	0.78	5.17	1.18	0.83	0.26^**^	0.31^**^	0.30^**^	–	0.36^**^	0.36^**^	0.35^**^	0.35^**^	0.29^**^	0.38^**^	0.31^**^
CommitteeLM	5.86	0.77	0.85	5.82	0.78	0.84	0.61^**^	0.45^**^	0.51^**^	0.32^**^	–	0.46^**^	0.47^**^	0.56^**^	0.49^**^	0.46^**^	0.54^**^
MeetingMgt	6.06	0.76	0.74	5.96	0.80	0.76	0.46^**^	0.37^**^	0.49^**^	0.35^**^	0.52^**^	–	0.54^**^	0.46^**^	0.37^**^	0.50^**^	0.43^**^
InfoMgt	5.56	0.94	0.82	5.60	0.96	0.83	0.50^**^	0.35^**^	0.52^**^	0.42^**^	0.50^**^	0.58^**^	–	0.60^**^	0.41^**^	0.54^**^	0.46^**^
RiskCompM	5.63	0.87	0.86	5.61	0.91	0.88	0.49^**^	0.42^**^	0.41^**^	0.39^**^	0.56^**^	0.49^**^	0.64^**^	–	0.53^**^	0.57^**^	0.50^**^
RoleClarity	5.35	1.08	0.77	5.29	1.15	0.80	0.35^**^	0.54^**^	0.39^**^	0.34^**^	0.52^**^	0.43^**^	0.46^**^	0.54^**^	–	0.43^**^	0.42^**^
StratDirectn	5.66	0.90	0.81	5.59	0.99	0.81	0.56^**^	0.46^**^	0.50^**^	0.39^**^	0.54^**^	0.56^**^	0.58^**^	0.59^**^	0.54^**^	–	0.49^**^
PfmceMgt	5.49	0.92	0.59	5.39	1.00	0.65	0.59^**^	0.43^**^	0.57^**^	0.30^**^	0.57^**^	0.45^**^	0.49^**^	0.49^**^	0.43^**^	0.52^**^	–

a* *EFA sub-sample (n = 842)*.

b* *CFA sub-sample (n = 704); EFA sub-sample correlations above the diagonal, CFA sub-sample correlations below the diagonal*;

** *p < 0.01*.

*Comms, effective communication and teamwork; SelfAssess, board self-assessment; ChairLdshp, chair leadership; RemMgt, remuneration management; CommitteeLM, committee leadership and management; MeetingMgt, meeting management record keeping; InfoMgt, information management; RiskCompM, risk and compliance management; RC, role clarity; StratDirectn, strategic direction; PfmceMgt, performance management of board members*.

## Discussion

The aim of the study was to validate an adapted version of Leblanc and Gillies' (2005) measure of board effectiveness. Given that there has been limited empirical validation of measures of Board effectiveness, the research contributes insights into the dimensions by which Board effectiveness can reliably be measured, and therefore monitored, managed and improved.

Validation of the measure was conducted on a large sample using both exploratory and confirmatory factor analytic procedures. The results identified 11 dimensions and, as such, support the arguments that board functioning is complex and multi-faceted (Nicholson and Kiel, 2004). In support of their content validity, the 11 dimensions largely correspond to those previously identified by academics and professional bodies (Kiel and Nicholson, 2005; Leblanc, 2005; Australian Institute of Company Directors, 2016). The 11 dimensions identified include: (1) effective internal communication and teamwork; (2) effective self-assessment; (3) effective leadership by the Chair: (4) effective committee leadership and management; (5) effective meeting management and record keeping; (6) effective information management; (7) clarity of board member roles and responsibilities; (8) effective internal performance management of members of the board; (9) effective oversight of strategic direction; (10) remuneration management; and (11) risk management and compliance. Table 3 shows that the 11 factors, to a large extent, correspond to Leblanc and Gillies' (2005) “how,” “do,” and “what” factors but did not replicate a clear “who” factor.

Six of the 11 factors corresponded exclusively or predominantly to Leblanc and Gillies (2005) “how” items, reflecting discrete process factors for how a board can operate effectively. Communication and teamwork refer to the degree to which the board works as a team, board members trust and respect each other, and board discussions are positive, productive and constructive. Board self-assessment relates to the degree to which the board assesses the effectiveness of the chair, committee chairs and individual directors. Leadership effectiveness of the chair refers to the way in which the chair effectively facilitates discussion, leads effective decision-making, role models positive leadership, and encourages constructive interpersonal dynamics and relationships between the members of the board. Information management refers to the degree to which board members perceive that important information (particularly from management) is presented to them in a timely, well-managed and appropriate manner. Committee leadership and management refers to the leadership role the committee chairs play in representing their committees at board meetings and the degree to which committee chairs effectively facilitate information sharing and foster positive working relationships with management. Meeting management refers the extent to which the board has administrative processes in place to effectively manage the board's agendas, meetings, minutes, and the distribution of written reports.

The three dimensions that most closely corresponded to Leblanc and Gillies' (2005) “do” factors included remuneration management; risk management and compliance; and oversight of strategic direction. Remuneration management refers to how effectively the board carries out its role with regards to setting appropriate remuneration packages for the CEO and management, and the degree to which remuneration is linked to the successful implementation of organizational strategy. Risk management and compliance refers to the extent to which the board perceives the organization as having effective internal control systems and procedures in place to identify risk factors, and to ensure they are brought to the attention of the board, and appropriately mitigated. Oversight of strategic direction refers to the degree to which the board has an understanding of the actions required to execute the organization's strategic plan.

The dimension that most closely corresponded to Leblanc and Gillies' (2005) “what” factor focused on establishing role clarity. The dimension refers to the extent the board has processes and documentation in place to clearly define the respective responsibilities of the board, the chair and committee chairs. The eleventh dimension consisted of an amalgam of LeBlanc and Gillies' who, what and how items, and refers to the extent the board sets clear expectations about performance and takes appropriate action when inappropriate or undesirable board behaviors are displayed.

The results of the current study suggest a revised, more differentiated and statistically defensible framework for evaluating board effectiveness. In contrast to the model and measures proposed by Leblanc and Gillies (2005), the results suggest 11 discrete dimensions of board functioning that can reliably be assessed using three to five items per dimension. Short reliable measures can be less burdensome for survey respondents because they require the investment of less time and effort (Rolstad et al., 2011; Schaufeli, 2017). Furthermore, rather than supporting broad and generic taxonomies such as “what, who, what and do” (Leblanc and Gillies, 2005) or “competencies, structures, and behaviors” (Beck and Watson, 2011), the results support the use of more fine-grained and differentiated dimensions. The relatively modest correlations between the 11 factors did not suggest they could usefully be grouped within more generic or higher-order factors (Christiansen et al., 1996).

As previously noted, the current findings to a large extant confirm or overlap with existing conceptualisations of board performance and functioning. Six of the dimensions broadly overlap with Leblanc and Gillies' five “how” factors. The communication and teamwork, role clarity, and strategic oversight factors largely correspond to Beck and Watson's 2011 “board competencies.” Similarly, board self-assessment, remuneration management, risk management and compliance, and information management align with Beck and Watson's “board behaviors.” Such overlap and alignment was expected, and provides for cross-validation of the importance and generalizability of the 11 dimensions identified. However, in contrast to existing models and measures, the present results are supported by quite rigorous statistical analyses.

### Practical Implications

The model presented in the current study suggests that boards are complex and multi-faceted and must focus on numerous diverse activities to work effectively. The development of a valid measure of board effectiveness has clear practical implications. A valid and reliable measure can be used with confidence internally by Board members to help them assess and improve their own functioning and effectiveness. The use of reliable measures enable confident assessments of whether self-evaluations change over time and in which direction. Similarly, external consultants can use the dimensions and the measure to audit board effectiveness and to diagnose where their client organizations can best develop and change. The large sample size and the variety of industries included in the current sample also lend confidence to the generalizability of the measure.

## Limitations

While the current study has provided new insights into important elements contributing to board effectiveness, some limitations need to be acknowledged. First, the results relied on self-report measures and as such are subject to the threat of “common method variance” (CMV). CMV refers to the variance in the proposed model that is a result of the measurement method, as opposed to true variance in the statistical model (Podsakoff et al., 2003). However, given that the measurement model demonstrated acceptable fit to the data, given that the correlations between the measured constructs were moderate and varied quite considerably, given the very modest average reduction in the standardized loadings after a common methods factor was included, and given that all the factor loadings remained statistically significant after the common methods factor was modeled, the issue of CMV appears not to be overly problematic. Nevertheless, future research could usefully incorporate multi-rater or longitudinal data points to help address the risk of CMV. Second, no objective data were collected concerning the financial performance of the organizations sampled. Thus, although the current model and measure suggests a range of dimensions that are important for effective Board functioning, their impact or success in delivering financial or other objective metrics could not be determined. Finally, given the modest reliability coefficient for the “Performance Management” dimension, additional items should be developed to more precisely define the construct.

### Considerations for Future Research

As noted above, future research could usefully be directed toward validating the identified dimensions of effective board functioning against objective criteria. Previous research has shown positive associations between board practices and organizational performance (e.g., Drobetz et al., 2004), so determining the relative contributions of the 11 factors to such performance would be instructive. Subjective outcome measures of board satisfaction and performance could also usefully be developed and validated. Future research might then employ multi-level model perspectives and methods to better capture the amount of variance in outcomes that can be attributed to the boards themselves as opposed to the variance that can be attributed to individuals across all boards. Additional research could also be designed that draws more closely from the team effectiveness literature to better understand how boards function and the impact on overall business performance. Payne et al. (2009), for example, found that team-based attributes and practices were associated with increased financial performance assessed in terms of return on assets (ROA), earnings per share (EPS), and return on sales (ROS).

It is widely acknowledged that boards are under increasing pressure to ensure sustained organizational success and competitive advantage (Buchwald and Thorwarth, 2015). Dimensions of board functioning might therefore usefully focus on Board member competence and capability with respect to understanding the implications of new technologies and more agile ways of working. Future researchers will hopefully be able to develop valid and reliable measures of such dimensions that build on the 11 factor model presented in this study.

Overall, future research is needed to establish with confidence whether the psychometrically defensible measures reported here are indeed associated with, or predictive of, performance outcomes. The present results suggest that using the dimensions and measures described, if reliable outcome measures can be accessed, then such future research can be conducted and interpreted with confidence.

## Conclusion

This study aimed to validate an adapted version of Leblanc and Gillies' (2005) measure of board effectiveness. Overall, the results supported a multidimensional conceptualization of board effectiveness. Eleven discrete dimensions were identified that will help boards and other stakeholders reliably assess the level of board functioning. This research makes significant contributions to the current corporate governance literature, as it presents a large sample empirically-validated measure that can be used with confidence in organizational settings.

## Author Contributions

SA, SLA, MD, and NB all contributed substantially to the conception and design of the research, interpretation of the findings, and preparation of the manuscript. SA and SLA contributed to the analysis of the data.

### Conflict of Interest Statement

The authors declare that the research was conducted in the absence of any commercial or financial relationships that could be construed as a potential conflict of interest.
